# Harnessing CRISPR/Cas9 for Enhanced Disease Resistance in Hot Peppers: A Comparative Study on *CaMLO2*-Gene-Editing Efficiency across Six Cultivars

**DOI:** 10.3390/ijms242316775

**Published:** 2023-11-26

**Authors:** Jae-Hyeong Park, Hyeran Kim

**Affiliations:** 1 Interdisciplinary Graduate Program in BIT Medical Convergence, Kangwon National University, Chuncheon 24341, Republic of Korea; wogud4601@kangwon.ac.kr; 2 Department of Biological Sciences, College of Natural Sciences, Kangwon National University, Chuncheon 24341, Republic of Korea

**Keywords:** CRISPR/Cas9 RNP, genome editing, *CaMLO2*, pepper leaf protoplasts, *Capsicum annuum*, commercial hot pepper cultivars

## Abstract

The *Capsicum annuum* Mildew Locus O (*CaMLO2*) gene is vital for plant defense responses against fungal pathogens like powdery mildew, a significant threat to greenhouse pepper crops. Recent advancements in genome editing, particularly using clustered regularly interspaced short palindromic repeats (CRISPR)/Cas9, have unlocked unprecedented opportunities for modifying disease-resistant genes and improving crop characteristics. However, the application of CRISPR technology in pepper cultivars has been limited, and the regeneration process remains challenging. This study addresses these limitations by investigating the feasibility of using the validated *CaMLO2* genetic scissors system in six commercial hot pepper cultivars. We assessed the gene-editing efficiency of the previously reported high-efficiency Cas9/*CaMLO2*single-guide RNA (sgRNA)1-ribonucleoprotein (RNP) and the low-efficiency Cas9/*CaMLO2*sgRNA2-RNP systems by extending their application from the bell pepper ‘Dempsey’ and the hot pepper ‘CM334’ to six commercial hot pepper cultivars. Across the six cultivars, *CaMLO2*sgRNA1 demonstrated an editing efficiency ranging from 6.3 to 17.7%, whereas *CaMLO2*sgRNA2 exhibited no editing efficiency, highlighting the superior efficacy of sgRNA1. These findings indicate the potential of utilizing the verified Cas9/*CaMLO2*sgRNA1-RNP system to achieve efficient gene editing at the *CaMLO2* locus in different *Capsicum annuum* cultivars regardless of their cultivar genotypes. This study provides an efficacious genome-editing tool for developing improved pepper cultivars with *CaMLO2*-mediated enhanced disease resistance.

## 1. Introduction

The genus *Capsicum* has been domesticated in the American tropics with breeding programs for human consumption [1]. The *C. annuum* species, which includes three closely related forms (*C. annuum*, *C. chinense*, and *C. frutescens*), is a globally consumed horticultural crop with culinary and medicinal significance. However, it is susceptible to climate-induced proliferation of pathogens and vectors [2]. Climate change has exacerbated powdery mildew, a disease that inhibits pepper growth and yield by disrupting photosynthesis and hormonal production. Wind and water aid in the spreading of powdery mildew infections [3,4]. In addressing the vulnerability of peppers to powdery mildew, there has been a growing focus on utilizing clustered regularly interspaced short palindromic repeats (CRISPR)/Cas9 editing of the Mildew Locus O (*MLO*) gene, which plays a pivotal role in enhancing resistance to powdery mildew [5,6]. Our preceding studies have shown precise editing of the *C. annuum MLO* gene (*CaMLO2*) via CRISPR in both ‘Dempsey’ and ‘CM334’ pepper protoplasts, a stride toward powdery mildew resilience and crop enhancement [5,6]. Pepper molecular breeding is of significant importance due to its potential to revolutionize pepper cultivation by enhancing crop yield, quality, and resilience. The advanced breeding approach utilizes insights from molecular genetics and genomics, combining scientific understanding with practical agriculture to accelerate the development of improved pepper cultivars that are more productive, resilient, and better aligned with consumer preferences. This has the potential to transform pepper cultivation, address agricultural challenges, and contribute to food security and economic prosperity.

Recent strides in genome-editing technology offer exceptional ways to modify DNA sequences precisely in living organisms [7,8]. Genome-editing tools, like zinc finger nucleases (ZFNs), transcription activator-like effector nucleases (TALENs), and CRISPR/Cas9 systems, have enabled refined gene editing in plants [9]. Among these, CRISPR/Cas9 stands out with its proficiency, precision, and adaptability [7]. SpCas9 from *Streptococcus pyogenes* is a widely applied Cas9 nuclease, guided by single-guide RNAs (sgRNAs) to target specific DNA sequences and accomplish precise genetic modifications through cellular repair mechanisms. Its broad utility and effectiveness in genetics and biotechnology have been well established [10,11]. The simplicity and cost-effectiveness of CRISPR/Cas9 tools have led to its extensive adoption in various organisms, including plants [12,13,14,15,16,17,18]. Its application in crop genome editing, particularly in genes governing growth, fertility, and disease resistance, holds promise [19,20,21,22].

Plant protoplasts, distinct derivatives of plant cells, harbor multifaceted potential for a wide range of applications [23]. In agriculture, their perpetual culturing confers a noteworthy edge, facilitating the generation of superior crop variants via seed production or large-scale vegetable cultivation [24]. In biological research, protoplasts offer invaluable tools for exploring plant physiology, genetics, and biochemistry [25,26,27,28]. Genetic studies, particularly concerning gene functions and mutational effects, rely on protoplasts [23,29]. Notably, protoplast fusion has emerged as a potent technique enabling hybrid plant creation and genetic material transfer between diverse plant species. Protoplast fusion plays a pivotal role in plant breeding, extending opportunities to enhance traits and introduce novel characteristics [29,30]. Within the field of gene editing, the use of CRISPR/Cas9 ribonucleoprotein (RNP) delivery to plant protoplasts holds significant promise for high-efficiency genome editing [29]. Delivery of CRISPR/Cas9 RNPs to protoplasts can be accomplished through a polyethylene glycol (PEG) treatment, gene gun, or electroporation [29,31,32]. These methods exhibit exceptional gene-editing efficiency not only in the model plant *Arabidopsis thaliana* but also in crop protoplasts, such as lettuce, soybean, petunia, camelina, and pepper [5,29,33,34,35].

*C. annuum* encompasses bell pepper varieties known for their mild and sweet flavor and hot pepper varieties characterized by pronounced spiciness, reflecting the diverse genetic traits found within various cultivars of this species [36]. Previously, we identified a high-efficiency CRISPR/Cas9 RNP complex targeting the *CaMLO2* gene in the hot pepper ‘CM334’ and the bell pepper ‘Dempsey’ [5]. However, it remains undetermined whether the Cas9 RNP can be broadly applied across a range of pepper cultivars with varied genetic profiles. To further explore the prospect for wide-ranging resistance to powdery mildew through leveraging this CRISPR/Cas9 RNP complex, we employed six commercial hot pepper cultivars with different horticultural characteristics. Our findings from the extended analyses of the efficiency and specificity of the CRISPR/Cas9 RNP across different commercial cultivars will furnish valuable insights, paving the way for the applicability of the CRISPR/Cas9 RNP tool for future molecular pepper breeding programs.

## 2. Results

### 2.1. Robust Hot Pepper Protoplasts Isolated from Six Commercial Cultivars

**Figure 1:**
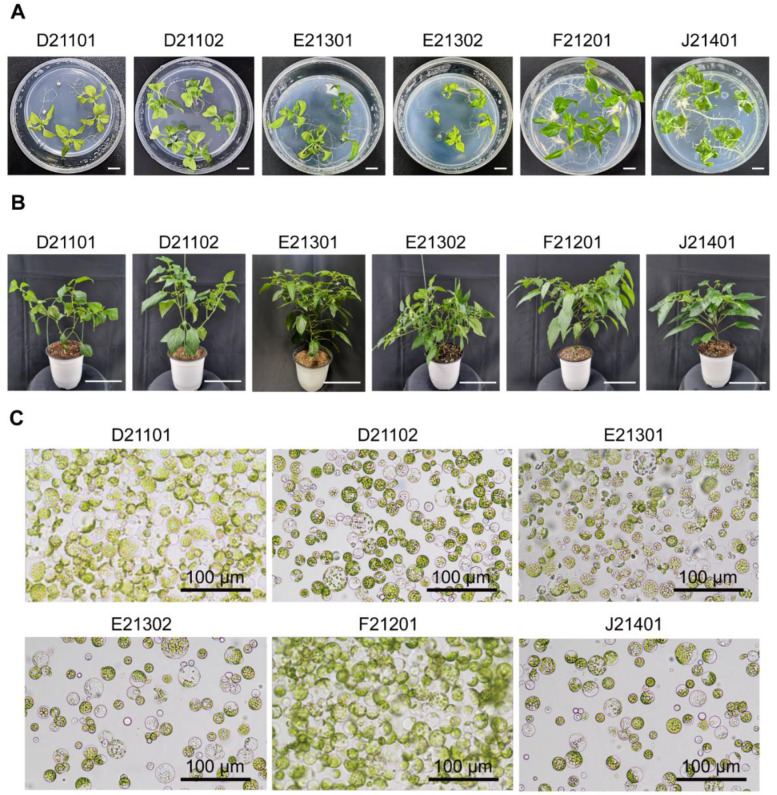
Observations of the six commercial hot peppers in *Capsicum annuum* and their leaf-derived protoplasts. (**A**) Six MS media-grown hot pepper cultivars at five weeks old (scale bars = 1 cm). (**B**) Soil-transferred hot pepper cultivars at different developmental stages at ten weeks old (scale bars = 10 cm). (**C**) Five-week-old pepper leaf-derived protoplasts.

To extend DNA-free CRISPR/Cas9-mediated genome editing in peppers, we explored the following six commercial hot pepper cultivars: D21101, D21102, E21301, E21302, F21201, and J21401. These six pepper cultivars have different agronomic traits, as outlined in Table 1, encompassing factors such as germplasm origin, pungency level, resistance to various pathogens, and growing purpose. Despite these varied traits, none of the six cultivars have powdery mildew resistance (personal communication with the seed provider). Careful observation was conducted on these cultivars at two distinct growth phases: a five-week in vitro stage (Figure 1A) and a subsequent ten-week stage where they were nurtured in soil (Figure 1B). Notably, these cultivars exhibited variations in their growth dynamics. The six cultivars produced their third leaves for isolating protoplasts during the five-week growing period. Even though the six pepper cultivars belong to *C. annuum*, we examined whether these six pepper cultivars produced robust and stable protoplasts that could be used with the DNA-free CRISPR/Cas9 RNP. Upon employing cell-wall digesting enzymes to extract protoplasts from the six pepper cultivars, we noted an absence of substantial differences in the general appearance of the resultant protoplasts (Figure 1C). Therefore, we delivered DNA-free CRISPR/Cas9 RNP into the resilient pepper protoplasts derived from each of the assessed six cultivars.

### 2.2. The Conserved CaMLO2 Gene among Six Commercial Hot Peppers

**Figure 2:**
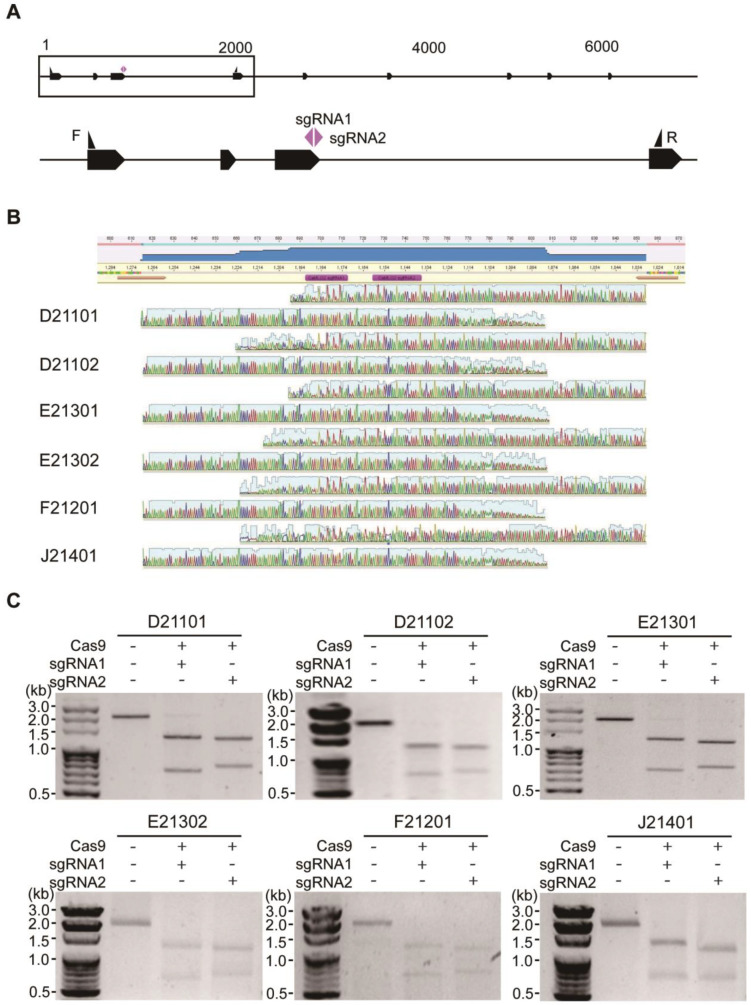
Evaluation of the *CaMLO2* target gene and the two designated CRISPR/Cas9 RNP complexes in the six commercial pepper cultivars. (**A**) Target locus of the *CaMLO2* gene, the two designed sgRNAs for Cas9, and the specific primer pair. (**B**) Target sequence confirmation of the *CaMLO2* genetic locus of the six pepper cultivars by Sanger sequencing. The specific primers (brown marks) were designed to target the third exon of *CaMLO2* containing both sgRNA1 and sgRNA2. The magenta marks designate sgRNA1 and sgRNA2 of *CaMLO2*. (**C**) In vitro cleavage assay with preassembled Cas9 only (as the control), Cas9-sgRNA1, and Cas9-sgRNA2 for the *CaMLO2* gene in the six cultivars.

Although the gene structure of the *MLO* homologs is highly conserved as membrane-anchored by seven transmembrane helices with an extracellular N-terminus and an intracellular C-terminus, the *MLO* family members are genetically diverse [37]. The target sequence specificity of the CRISPR tools critically impacts the efficiency of the target gene editing in a genome. We investigated the genetic loci of the *CaMLO2* gene within the six pepper cultivars using a gene-specific primer pair (Table S1) previously validated in the bell pepper ‘Dempsey’ and the hot pepper ‘CM334’ (Figure 2A) [5]. The PCR-amplified 2.0 kb *CaMLO2* genomic regions from the six cultivars were analyzed by Sanger sequencing, and the six amplicon sequences were aligned to the reference *CaMLO2* gene. All the sequenced genetic regions among the six cultivars were identical to the reference *CaMLO2* sequence without a single nucleotide polymorphism and contained the previously designated *CaMLO2*sgRNA1 and sgRNA2 regions (Figure 2B).

With the confirmation of the target *CaMLO2* gene sequences in all six pepper cultivars, we conducted in vitro cleavage analyses to verify whether the preassembled CRISPR/Cas9-sgRNA1 or -sgRNA2 RNP complexes could specifically cleave the target *CaMLO2* gene throughout the six commercial cultivars. Based on the validated conservation of the *CaMLO2* gene sequences, the amplified approximately 2 kb fragments of target DNA were cleaved into two fragments of approximately 1.2 and 0.8 kb (Figure 2C) in all six commercial hot pepper cultivars, as described in a previous study using the reference cultivars [5]. These results suggest that the preassembled DNA-free CRISPR/Cas9-sgRNA1 or -sgRNA2 RNP complexes could be used to generate *CaMLO2*-edited peppers throughout the six commercial hot pepper cultivars.

### 2.3. Comparison of Indel Frequencies of CaMLO2sgRNA1 or sgRNA2 among the Six Commercial Hot Peppers

**Figure 3:**
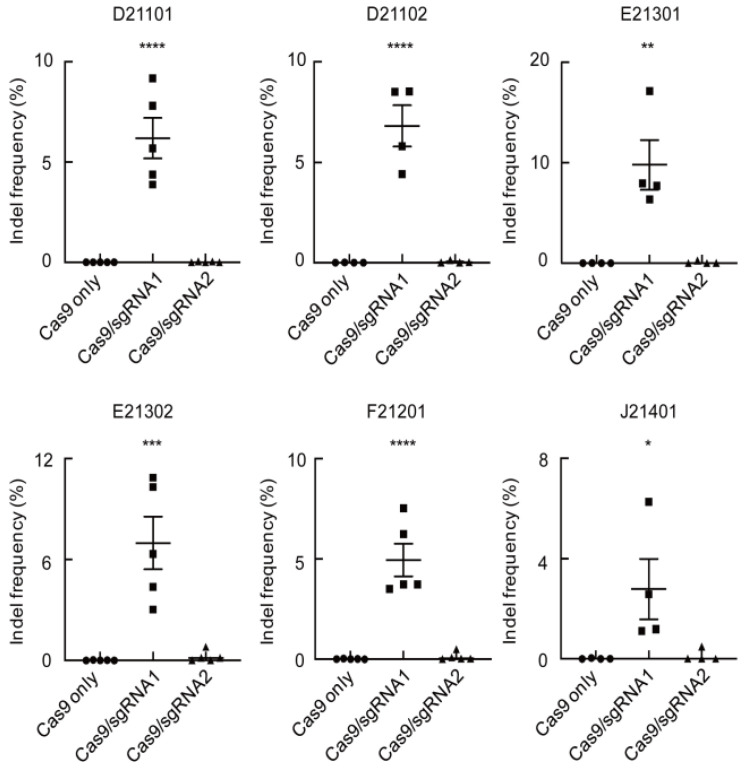
Comparison of the Indel frequencies (%) with Cas9 only, Cas9-sgRNA1, or Cas9-sgRNA2 transfected into the protoplasts of the six cultivars. The vertical bars represent the mean ± standard deviation (*n* ≥ 3). The Indel frequencies (%) were calculated as the number of measured reads divided by the number of total reads. In the graph, the rotundity, square, and triangle shapes depict the Indel frequencies corresponding to Cas9 only, Cas9-sgRNA1, and Cas9-sgRNA2 transformed protoplasts, respectively. Each feature is a biological replicate. An asterisk indicates a significant difference compared with Cas9 only, based on the one-way ANOVA; ****, *p* < 0.0001; ***, *p* < 0.001; **, *p* < 0.01; *, *p* < 0.05.

To investigate precise *CaMLO2* gene editing within the six-pepper protoplast-based transient systems, we conducted in vivo protoplast-based CRISPR/Cas9 RNP delivery experiments on the six commercial hot pepper cultivars. All the CRISPR/Cas9 RNP-mediated gene-editing experiments were conducted with at least four to five biological replicates (Figure 3). In the D21101 cultivar, insertions/deletions (Indels) were observed at the target sites with frequencies that ranged from 3.9 to 9.2% for Cas9/*CaMLO2*sgRNA1 and from 0.0 to 0.2% for Cas9/*CaMLO2*sgRNA2. In the D21102 cultivar, Indels were observed at frequencies that ranged from 4.4 to 8.5% for Cas9/*CaMLO2*sgRNA1 and from 0.0 to 0.1% for Cas9/*CaMLO2*sgRNA2. The E21301 cultivar had Indel frequencies that ranged from 6.3 to 17.7% for Cas9/*CaMLO2*sgRNA1 and 0.0 to 0.3% for Cas9/*CaMLO2*sgRNA2. The E21302 cultivar had Indel frequencies that ranged from 3.0 to 10.9% for Cas9/*CaMLO2*sgRNA1 and from 0.0 to 0.8% for Cas9/*CaMLO2*sgRNA2. The F21201 cultivar had Indel frequencies that ranged from 3.5 to 7.5% for Cas9/*CaMLO2*sgRNA1 and from 0.0 to 0.2% for Cas9/*CaMLO2*sgRNA2. The J21401 cultivar had Indel frequencies that ranged from 1.1 to 6.3% for Cas9/*CaMLO2*sgRNA1 and from 0.0 to 0.5% for Cas9/*CaMLO2*sgRNA2. In a one-way ANOVA conducted based on the Indel frequencies of all the pepper cultivars, a significant difference was observed in the Indel frequency for Cas9/*CaMLO2*sgRNA1. Specifically, the *p*-values for D21101, D21102, and F21201 were found to be below 0.0001, indicating a highly significant difference with Cas9 only and *CaMLO2*sgRNA2. These results indicate that the Indel frequencies of Cas9/*CaMLO2*sgRNA1 complexes were consistently higher than Cas9/*CaMLO2*sgRNA2 within the six commercial cultivars (Figure 3). These results demonstrate that Cas9/*CaMLO2*sgRNA1 complexes induce Indel mutations more efficiently than Cas9/*CaMLO2*sgRNA2 in the six tested hot pepper cultivars.

### 2.4. Comparison of the Indel Patterns by CaMLO2sgRNA1 or sgRNA2 among the Six Commercial Hot Peppers

**Figure 4:**
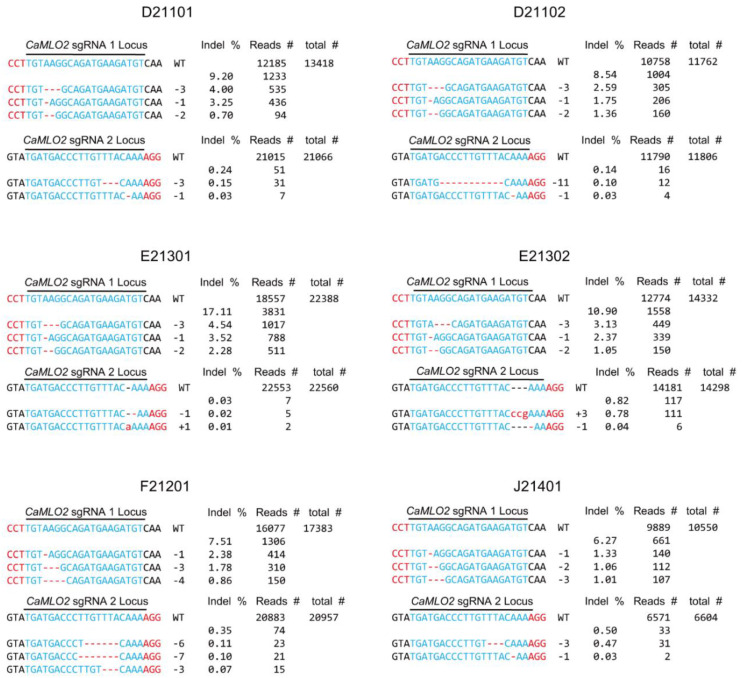
Analyses of the Indel patterns at the *CaMLO2* genetic locus in the Cas9-sgRNA1- or Cas9-sgRNA2-transformed leaf protoplasts of the six commercial hot peppers. These Indel patterns are listed with the top-three-ranked reads from those with the highest Indel frequencies among the biological replicates for each cultivar. The Indel frequencies (%) were calculated as the number of measured reads divided by the number of total reads. Total reads (#) were obtained by targeted deep sequencing. WT denotes the depicted target gene carrying the sgRNA sequences; Indel %, Indel frequencies of the edited reads; red letters, the PAM sequences; blue letters, CRISPR target (sgRNA) sequence; red hyphens (-), deleted nucleotides; red letters (+), inserted nucleotides.

In addition to the Indel frequencies, we also analyzed the Indel patterns at the target loci of the *CaMLO2* gene by targeted deep sequencing. The predominant Indel patterns induced by the Cas9/*CaMLO2*sgRNA1 complex were observed with high ranks as various nucleotide deletions ranging from one to nine nucleotides across all biological replicates from the six hot pepper cultivars (Figure 4 and Figure S1). Notably, several nucleotides deletions (−1, −2, −4, −5, −7, and −8) and one nucleotide insertion (+1) occurring immediately following the PAM sequence (5′-CCT-3′) resulted in the formation of an early stop codon within the third exon of *CaMLO2*. The deletion of three nucleotides (−3) generated a single amino acid deletion of the CaMLO2 protein. The Cas9/*CaMLO2*sgRNA2 delivered to the pepper protoplasts also showed various deletion patterns, from one nucleotide to eleven nucleotides and insertions of one or three nucleotides (Figure 4). These results indicate that the predominant Indel patterns induced by the Cas9/*CaMLO2*sgRNA1 complex successfully produce *CaMLO2* mutations in the applied six cultivars. Thus, the Cas9/*CaMLO2*sgRNA1 complex will be an excellent gene-editing tool for commercial pepper cultivars.

### 2.5. In Vivo Off-Taget Validation of the CaMLO2sgRNA1 Delivered across the Six Cultivars

**Figure 5:**
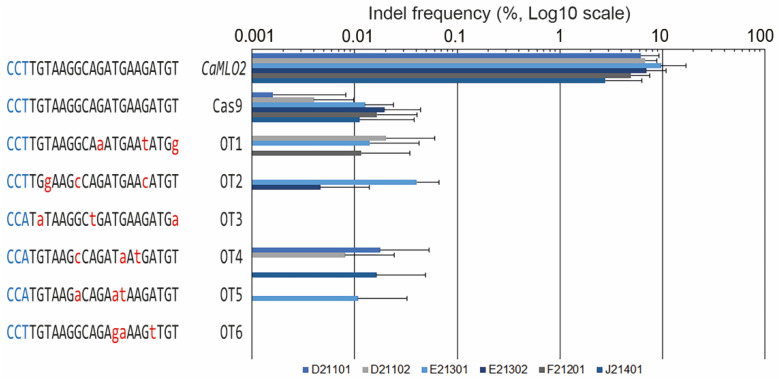
In vivo evaluation of *CaMLO2*sgRNA1 activity at potential off-target sites across the six pepper genomes. The Indel frequencies (%) at six candidate off-target (OT) sites (with three bp mismatches relative to the *CaMLO2*sgRNA1) were measured and validated in Cas9-sgRNA1 pepper protoplasts by targeted deep sequencing. Error bars represent the standard deviation (*n* ≥ 3); red letters, mismatched nucleotide bases; blue letters, PAM sequences.

To validate the specificity of the Cas9/*CaMLO2*sgRNA1 complex-mediated genome editing, we surveyed the pepper genomes in silico using the Cas-OFFinder program (http://rgenome.net, accessed on 20 November 2023). Initially, to avoid off-target effects, we specifically selected sgRNA1 and sgRNA2 that did not have two nucleotide mismatches based on the entire homology of the pepper reference genome, except the target sites, using Cas-Designer (http://rgenome.net, accessed on 20 November 2023). Therefore, we identified potential off-target (OT) sites with three nucleotide variations (Figure 5). We designed specific primer sets (Table S2) to amplify the putative OT loci from the genomic DNA isolated from the Cas9/*CaMLO2*sgRNA1 complex protoplasts across the six hot pepper cultivars. We performed targeted deep sequencing analyses from OT1 to OT6. No Indel mutations were detected at the six examined OTs compared with the Cas9-only protoplasts from the six cultivars (Figure 5). This finding suggests that the Cas9/*CaMLO2*sgRNA1 complex did not tolerate three nucleotide mismatches. Consequently, *CaMLO2*sgRNA1 demonstrated high sequence specificity, adequately targeting the *CaMLO2* target locus across the six cultivars. These results align with the previous findings of precise editing in tobacco and soybeans [33], indicating that Cas9/*CaMLO2*sgRNA1 can serve as an excellent gene-editing tool for precisely improving commercial pepper cultivars.

## 3. Discussion

We investigated the potential application of the *CaMLO2* genetic scissors, a DNA-free CRISPR/Cas9 RNP, to six commercial hot pepper cultivars. The six inbred hot pepper cultivars have different horticultural traits and growth rates. In the five-week in vitro stage, the F21201 cultivar grew nearly twice as tall as the E21302 cultivar (Figure 1A). However, these growth rates were less pronounced when grown in soil pots in the growth room (Figure 1B). The six commercial pepper cultivars used in this study exhibited typical characteristics of *C. annuum*-derived protoplasts (Figure 1C), similar to the *C. annuum*-derived protoplasts usually used in genome-editing studies, such as the hot pepper ‘CM334’ and the bell pepper ‘Dempsey’ [5]. However, unlike pure protoplasts from the hot pepper ‘CM334,’ the protoplasts isolated from the six hot pepper cultivars were stable enough for PEG-mediated, transient delivery of the DNA-free CRISPR/Cas9 RNP for editing the *CaMLO2* gene within 48 h. Notably, we further observed the variable viability among the six hot pepper cultivar-derived protoplasts over three days of cultivation, indicating distinct characteristics at the cellular level.

Before applying the CRISPR/Cas9 RNPs to the six hot pepper cultivar-derived protoplasts, we conducted a Sanger sequencing analysis to confirm the target gene loci of the designed *CaMLO2*sgRNA1 and sgRNA2 based on the reference genomes of the hot pepper ‘CM334’ and the bell pepper ‘Dempsey.’ Thus, the genetic loci of the six cultivars were precisely conserved at the target sgRNA1 and sgRNA2 for the *CaMLO2* gene, like the two reference peppers [5]. Subsequently, we performed in vitro DNA cleavage experiments to validate the activities of the CRISPR/Cas9 RNP complexes with *CaMLO2*sgRNA1 or *CaMLO2*sgRNA2. The cleavage activities of the Cas9sgRNA1 or Cas9sgRNA2 complexes exhibited an elaborate function at the *CaMLO2* target amplicon of the genomes of the six hot peppers (Figure 2C).

Since the whole genome sequence of the hot pepper ‘CM334’ and the bell pepper ‘Dempsey’ has been reported [38,39], both ‘CM334’ and ‘Dempsey’ are genome-editable cultivars using the CRISPR/Cas9 and CRISPR/Cpf1 tools [5,6]. Moreover, the DNA-free CRISPR/Cas9 RNP tool-mediated gene editing in both pepper protoplasts showed far better editing efficiency than in *Agrobacterium*-mediated transformation using pepper callus [5,6]. Using CRISPR/RNPs provides a method of minimizing off-target effects and cytotoxicity related to DNA transfection while simultaneously avoiding the potential integration of small DNA fragments from the plasmids [33]. Our extensive off-target analyses across all six cultivars provided evidence that the Cas9/*CaMLO2*sgRNA1 genetic scissors are effective across diverse cultivars and serve as an elaborate gene-editing tool, displaying an intolerance to three mismatches. This study expanded on the fact that pepper protoplast-based genome editing of seven hot pepper cultivars (CM334, D21101, D21102, E21301, E21302, F21201, and J21401) and a bell pepper cultivar (Dempsey) is a good strategy for developing an improved new pepper cultivar.

Although the evaluation of anticipated powdery mildew resistance resulting from the edited pepper cultivars with Cas9/*CaMLO2*sgRNA1 remains to be validatednot shown, the assessment of disease resistance through editing *MLO* genes has been demonstrated in various plants. Previous studies have documented that powdery mildew resistance can be attained through *MLO* gene mutation in barley [40]. Other studies extended the understanding of powdery mildew resistance to model plants such as *Arabidopsis* and rice [41,42]. Notably, gene functional studies have successfully revealed biotic stress resistance through the *MLO* gene in peppers using RNAi [43]. Moreover, in recent gene-editing studies, CRISPR/Cas9 systems have successfully conferred resistance to powdery mildew in hexaploid bread wheat [44,45]. Hence, editing *CaMLO2*, the ortholog of the *MLO* gene in peppers, could confer resistance to powdery mildew, similar to the demonstrated effects observed in various plant species.

However, one issue is the need for a robust regeneration protocol for pepper protoplast-based whole plants because the cultivation of pepper protoplasts primarily relies on protocols for a few cultivars, such as Dulce Italiano [46] and California Wonder [47]. There is a need to establish significantly better regeneration methods for various cultivars, especially those whose whole genome sequence is available. Fortunately, we have recently updated a reliable regeneration method for the pepper cultivar ‘Dempsey’ [48]. Therefore, successfully editing the protoplasts from eight cultivars would be a good resource for initiating a molecular breeding program to regenerate whole pepper plants besides the effort of *Agrobacterium*-mediated transformation in peppers.

We confirmed that the highly efficient *CaMLO2* genetic scissors, CRISPR/Cas9 *CaMLO2*sgRNA1 RNP, could be successfully applied to six commercial hot peppers regardless of their genotypes and agricultural traits. Moreover, these results can serve as a foundation for developing genetically edited peppers by demonstrating the versatility of validated gene-editing scissors and identifying candidate cultivars advantageous in post-transformation regeneration processes. This study will contribute to expanding the knowledge and understanding of genome-editing technology in pepper genetics and will provide a valuable tool for developing improved pepper cultivars with enhanced disease resistance and other desirable traits.

## 4. Materials and Methods

### 4.1. Plant Materials

Six cultivars (D21101, D21102, E21301, E21302, F21201, and J21401) of *C. annuum* were provided by the New Breeding Technology Center (Gwangju, Republic of Korea). The seeds of the pepper cultivars were sterilized with 70% ethanol and 2% commercial bleach and washed five times with distilled water for one minute each. The surface-sterilized seeds were germinated on a medium composed of half-strength Murashige and Skoog (MS) with MES (M0222, Duchefa Biochemie, Haarlem, The Netherlands; M1053, Duchefa Biochemie, Haarlem, The Netherlands), 3% sucrose, and 0.8% phytoagar (P1003, Duchefa Biochemie, Haarlem, The Netherlands). The medium was then adjusted to a pH of 5.8. The sowed seeds were incubated in the dark at 25 °C for a week. The germinated pepper seedlings were grown at 25 ± 2 °C, with a 16 h light and 8 h dark photoperiod, and 60% relative humidity in a growth chamber for four weeks or transferred into soil pots in a growth room. The images of the cultivars in soil pots were taken when they were ten weeks old.

### 4.2. CaMLO2 Genomic Loci Analyses by Sanger Sequencing

Genomic DNA from the six pepper cultivars was prepared using a Plant SV Mini kit (GeneAll, Seoul, Republic of Korea). We performed a PCR using specific primers targeting the third exon sequence of *CaMLO2*, the target region of the designed guide RNAs (Table S1). The target amplicons were sequenced by Sanger sequencing to confirm the nucleotide sequences and the designated two sgRNAs at the genetic loci of *CaMLO2*.

### 4.3. Preparation of the Single-Guide RNA and Cas9 Protein

The two single-guide RNAs (sgRNA) for *CaMLO2* gene editing have been reported in our previous studies [5,6]. The sgRNAs were synthesized by in vitro transcription using T7 RNA polymerase (New England Biolabs, MA, USA), purified using an Exipin PCR SV kit (GeneAll, Seoul, Republic of Korea) and dissolved in DEPC-treated water. *E. coli* Rosetta harboring the His-tagged Cas9 expression vector was cultured in 1 mM isopropyl-b-D-thiogalactoside (IPTG) and harvested by centrifugation. The sonicated total protein extract was purified using Ni-NTA affinity chromatography (Ni-NTA agarose, Qiagen, Hilden, Germany) [33].

### 4.4. In Vitro Cleavage Analysis Using the Designed CRISPR/Cas9 RNP

The activity of the designed sgRNA and purified Cas9 was validated by an in vitro cleavage analysis. The target DNA amplicons were generated by the primer pair of F0 and R0 (Table S1) and digested with a mixture of purified Cas9 and sgRNA1 or sgRNA2 in 10X NEB 3.1 buffer for one hour at 37 °C and subsequently incubated with RNase A for 30 min at 37 °C. The digested target DNA amplicons were confirmed by agarose gel electrophoresis.

### 4.5. Protoplast Isolation and PEG-Mediated Transfection

Pepper leaves of the six cultivars at five weeks old were digested in 1× VCP enzyme [49] for four hours at room temperature to isolate pure protoplasts. The digested pepper protoplasts were diluted with an equal volume of W5 solution (154 mM NaCl, 125 mM CaCl_2_, 5 mM KCl, 5 mM glucose, 1.5 mM MES-KOH, pH 5.6). The isolated protoplasts were gently collected by a low-speed swing centrifugation and then rinsed twice with the W5 solution. The isolated pepper protoplasts were counted using a hemocytometer. Approximately 2 × 10^5^ isolated protoplasts were used for the PEG-mediated delivery of the CRISPR/Cas9 RNP. Briefly, preassembled Cas9/sgRNA (1:6 molar ratio) complexes were carefully suspended with the counted protoplasts in 300 μL of MMG solution (400 mM mannitol, 15 mM MgCl_2_, 5 mM MES, pH 5.6). An equal volume of freshly prepared PEG solution (200 mM mannitol, 100 mM CaCl_2_, 40% PEG 4000) was added. The CRISPR/Cas9 RNP-delivered protoplasts were incubated at 25 °C for 48 h, harvested for genomic DNA extraction, and finally analyzed for target gene editing.

### 4.6. Targeted Deep Sequencing

The target gene locus was amplified by a nested PCR using specific primer pairs (F and R) and subsequently amplified using individual primary primer pairs (F1 and R1) to examine the Indel frequencies and patterns (Table S1). The six off-target loci were amplified by a nested PCR using specific primer pairs (OT F and OT R) and subsequently amplified using individual primary primer pairs (F1 and R1) to examine the Indel frequencies (Table S2). The target amplicons were attached with multiplexing indexes and specific sequencing adaptors by consecutive PCR. The amplicons were purified and sequenced using an Illumina MiSeq V2 Reagent Kit (300 cycle; San Diego, CA, USA). The raw data of paired-end MiSeq were analyzed using Cas-Analyzer (http://www.rgenome.net/cas-analyzer/#!, accessed on 20 November 2023) from the RGEN tools [50].

### 4.7. Statistical Analyses

The data are presented as the mean and standard deviation of at least three biological replicates. The significant difference (*, *p* < 0.05; **, *p* < 0.01, ***, *p* < 0.001, ****, *p* < 0.001) was assessed by a one-way ANOVA.

## 5. Conclusions

This study established the feasibility of robust protoplast generation and validated Cas9/*CaMLO2*sgRNA1 as an effective gene-editing tool without potential off-target effects in six commercial hot pepper cultivars. Despite the genetic diversity inherent across the six pepper cultivars, the examination of the genetic structure of the *CaMLO2* homologs revealed a consistent and reliable approach for targeting and modifying the gene. Furthermore, the analyses of Indel frequencies and patterns at the target locus of *CaMLO2* demonstrated the superiority of the Cas9/*CaMLO2*sgRNA1 complex in inducing effective *CaMLO2* mutations across the diverse set of the six cultivars. Consequently, Cas9/*CaMLO2*sgRNA1 emerges as a promising gene-editing tool with significant potential for enhancing genetic modifications in commercial pepper cultivars.

## Figures and Tables

**Table 1:** Summary of the agronomic traits of the six commercial hot peppers.

Cultivar Name	Agronomic Trait
D21101	Korean cultivar (KC) for pepper powder, highly pungent, resistant to Phytophthora blight
D21102	KC for pepper powder, highly pungent, resistant to Phytophthora blight, tomato spotted wilt virus (TSWV), and anthracnose
E21301	India cultivar for pepper powder
E21302	Southeast Asian cultivar, upright type, single-podded
F21201	KC for green pepper, highly pungent, resistant to Phytophthora blight
J21401	South American cultivar, jalapeño, resistant to bacterial leaf spot (BLS)

## Data Availability

All data supporting reported results can be found in the article.

## Supplementary Materials

The following supporting information can be downloaded at https://www.mdpi.com/article/10.3390/ijms242316775/s1.
