# Supplementary material for: Harnessing CRISPR/Cas9 for Enhanced Disease Resistance in Hot Peppers: A Comparative Study on CaMLO2-Gene-Editing Efficiency across Six Cultivars
Source: Int J Mol Sci. 2023 Nov 26;24(23):16775. doi: 10.3390/ijms242316775 (PMC10706117; doi:10.3390/ijms242316775)
Supplement: Supplementary file 1 [file ijms-24-16775-s001.zip › ijms-2703543-supplementary.pdf]

Harnessing CRISPR/Cas9 for Enhanced Disease Resistance in Hot Pepper: A Comparative Study on *CaMLO2*-Gene-Editing Efficiency across six cultivars

Jae-Hyeong Park[1] and Hyeran Kim[1, 2, *]

Supplementary Data

Table S1. Primers used in Sanger sequencing or targeted deep sequencing or sgRNA synthesis. The primary primer pair used for targeted deep sequencing was the same as that used for Sanger sequencing analysis.

Primer Name	Sequence
CaMLO2 F	5′-ATGGCTAAAGAACGGTCGAT-3′
CaMLO2 R	5′-ATGGAGCTGGTGTATTGCAT-3′
CaMLO2 F1 (primary F)	5′-TGGGATTCATATCATTGTTGTTG-3′
CaMLO2 R1 (primary R)	5′-CCGAATGTGTCTCAGCCTTT-3′
sgRNA1 (without PAM)	5′-ACATCTTCATCTGCCTTACA-3′
sgRNA2 (without PAM)	5′-TGATGACCCTTGTTTACAAA-3′

D21101

CaMLO2 sgRNA 1 Locus

```
     CCTTGTAAGGCAGATGAAGATGTCAA   WT
1st  CCTTGTA--GCAGATGAAGATGTCAA   -2
     CCTTGT-----AGATGAAGATGTCAA   -5
     CCTTGT-AGGCAGATGAAGATGTCAA   -1

2nd  CCTTGT----CAGATGAAGATGTCAA   -4
     CCTTGT---GCAGATGAAGATGTCAA   -3
     CCTTGT-AGGCAGATGAAGATGTCAA   -1

3rd  CCTTGT---GCAGATGAAGATGTCAA   -3
     CCTTGT-AGGCAGATGAAGATGTCAA   -1
     CCTTGT--GGCAGATGAAGATGTCAA   -2

4th  CCTTGT--GGCAGATGAAGATGTCAA   -2
     CCTTGT--------TGAAGATGTCAA   -8
     CCTTGT---GCAGATGAAGATGTCAA   -3

5th  CCTTGT--GGCAGATGAAGATGTCAA   -2
     CCTTGT---GCAGATGAAGATGTCAA   -3
     CCTTGT-AGGCAGATGAAGATGTCAA   -1
```

CaMLO2 sgRNA 2 Locus

```
     GTATGATGACCCTTGTTTACAAAAGG   WT
1st  GTATGATGACCCTTGT---CAAAAGG   -3
     GTATGATGACCCTTGTTTAC-AAAGG   -1
2nd  GTATGATGACCCTTGTTTAC-AAAGG   -1
```

D21102

CaMLO2 sgRNA 1 Locus

```
     CCTTGT-AAGGCAGATGAAGATGTCAA   WT
1st  CCTTGT--AGGCAGATGAAGATGTCAA   -1
     CCTTGTaAAGGCAGATGAAGATGTCAA   +1
     CCTTGT------AGATGAAGATGTCAA   -5

2nd  CCTTGT----GCAGATGAAGATGTCAA   -3
     CCTTGT--AGGCAGATGAAGATGTCAA   -1
     CCTTGT---GGCAGATGAAGATGTCAA   -2

3rd  CCTTGT----GCAGATGAAGATGTCAA   -3
     CCTTGT--AGGCAGATGAAGATGTCAA   -1
     CCTTGT---GGCAGATGAAGATGTCAA   -2

4th  CCTTGT---GGCAGATGAAGATGTCAA   -2
     CCTTG----------TGAAGATGTCAA   -9
     CCTTGT------AGATGAAGATGTCAA   -5
```

CaMLO2 sgRNA 2 Locus

```
     GTATGATGACCCTTGTTTACAAAAGG   WT
1st  GTATGATG-----------CAAAAGG   -11
     GTATGATGACCCTTGTTTAC-AAAGG   -1
```

E21301

CaMLO2 sgRNA 1 Locus

```
     CCTTGTAAGGCAGATGAAGATGTCAA   WT
1st  CCTTGT---GCAGATGAAGATGTCAA   -3
     CCTTGT-AGGCAGATGAAGATGTCAA   -1
     CCTTGT--GGCAGATGAAGATGTCAA   -2

2nd  CCTTGT-AGGCAGATGAAGATGTCAA   -1
     CCTTGT---GCAGATGAAGATGTCAA   -3
     CCTTGT--GGCAGATGAAGATGTCAA   -2

3rd  CCTTGT-AGGCAGATGAAGATGTCAA   -1
     CCTTGTA--GCAGATGAAGATGTCAA   -2
     CCTTGT---GCAGATGAAGATGTCAA   -3

4th  CCTTGT---GCAGATGAAGATGTCAA   -3
     CCTTGT-AGGCAGATGAAGATGTCAA   -1
     CCTTGT--GGCAGATGAAGATGTCAA   -2
```

CaMLO2 sgRNA 2 Locus

```
     GTATGATGACCCTTGTTTAC-AAAAGG   WT
1st  GTATGATGACCCTTGTTTAC--AAAGG   -1
     GTATGATGACCCTTGTTTACaAAAAGG   +1
```

E21302

CaMLO2 sgRNA 1 Locus

```
     CCTTGT-AAGGCAGATGAAGATGTCAA   WT
1st  CCTTGT----GCAGATGAAGATGTCAA   -3
     CCTTGT--AGGCAGATGAAGATGTCAA   -1
     CCTTGT---GGCAGATGAAGATGTCAA   -2

2nd  CCTTGTaAAGGCAGATGAAGATGTCAA   +1
     CCTTGT----GCAGATGAAGATGTCAA   -3
     CCTTGT-----CAGATGAAGATGTCAA   -4

3rd  CCTTGT----GCAGATGAAGATGTCAA   -3
     CCTTGT--AGGCAGATGAAGATGTCAA   -1
     CCTTGT---GGCAGATGAAGATGTCAA   -2

4th  CCTTGTaAAGGCAGATGAAGATGTCAA   +1
     CCTTGT-----CAGATGAAGATGTCAA   -4
     CCTTGT---GGCAGATGAAGATGTCAA   -2

5th  CCTTGTaAAGGCAGATGAAGATGTCAA   +1
     CCTTGT-----CAGATGAAGATGTCAA   -3
     CCTTG--------GATGAAGATGTCAA   -7
```

CaMLO2 sgRNA 2 Locus

```
     GTATGATGACCCTTGTTTAC---AAAAGG   WT
1st  GTATGATGACCCTTGTTTACccgAAAAGG   +3
     GTATGATGACCCTTGTTTAC----AAAGG   -1
2nd  GTATGATGACCCTTGTTTAC----AAAGG   -1
     GTATGATGACCCTTGTTTACa--AAAAGG   +1
3rd  GTATGATGAC---------C---AAAAGG   -9
     GTATGATGACCCTTGT---C---AAAAGG   -3
     GTATGATGACCCTTGTT------AAAAGG   -3
```

F21201

CaMLO2 sgRNA 1 Locus

```
     CCTTGT-AAGGCAGATGAAGATGTCAA   WT
1st  CCTTGT--AGGCAGATGAAGATGTCAA   -1
     CCTTGT----GCAGATGAAGATGTCAA   -3
     CCTTGT-----CAGATGAAGATGTCAA   -4

2nd  CCTTGT------AGATGAAGATGTCAA   -5
     CCTTGT-A--GCAGATGAAGATGTCAA   -2
     CCTTGT----GCAGATGAAGATGTCAA   -3

3rd  CCTTGT----GCAGATGAAGATGTCAA   -3
     CCTTGT--AGGCAGATGAAGATGTCAA   -1
     CCTTGT---GGCAGATGAAGATGTCAA   -2

4th  CCTTGT----GCAGATGAAGATGTCAA   -3
     CCTTGT--AGGCAGATGAAGATGTCAA   -1
     CCTTGT---GGCAGATGAAGATGTCAA   -2

5th  CCTTGT----GCAGATGAAGATGTCAA   -3
     CCTTGTaAAGGCAGATGAAGATGTCAA   +1
     CCTTGT---GGCAGATGAAGATGTCAA   -2
```

CaMLO2 sgRNA 2 Locus

```
     GTATGATGACCCTTGTTTAC-AAAAGG   WT
1st  GTATGATGACCCTTGTTTAC--AAAGG   -1
     GTATGATGACCCTTGTTTACaAAAAGG   +1
2nd  GTATGATGACCCT------C-AAAAGG   -6
     GTATGATGACCC-------C-AAAAGG   -7
     GTATGATGACCCTTGT---C-AAAAGG   -3
3rd  GTATGATGACCCTTGT---C-AAAAGG   -3
     GTATGATGACCCTTGTTTAC--AAAGG   -1
```

J21401

CaMLO2 sgRNA 1 Locus

```
     CCTTGT-AAGGCAGATGAAGATGTCAA   WT
1st  CCTTGT----GCAGATGAAGATGTCAA   -3
     CCTTGT---GGCAGATGAAGATGTCAA   -2
     CCTTGT--AGGCAGATGAAGATGTCAA   -1

2nd  CCTTGT---GGCAGATGAAGATGTCAA   -2
     CCTTGT------AGATGAAGATGTCAA   -5
     CCTTGT--AGGCAGATGAAGATGTCAA   -1

3rd  CCTTGTaAAGGCAGATGAAGATGTCAA   +1
     CCTTGT--AGGCAGATGAAGATGTCAA   -1
     CCTTGT---GGCAGATGAAGATGTCAA   -2

4th  CCTTGT--AGGCAGATGAAGATGTCAA   -1
     CCTTGT---GGCAGATGAAGATGTCAA   -2
     CCTTGT----GCAGATGAAGATGTCAA   -3
```

CaMLO2 sgRNA 2 Locus

```
     GTATGATGACCCTTGTTTACAAAAGG   WT
1st  GTATGATGACCCTTGT---CAAAAGG   -3
     GTATGATGACCCTTGTTTAC-AAAGG   -1
2nd  GTATGATGACCCTTGTTTAC-AAAGG   -1
```

Figure S1. The top 3-ranked *CaMLO2* editing patterns of all biological replicates of six commercial hot pepper cultivars. Red letter, PAM sequences; blue letter, CRISPR target (sgRNA) sequence; red hyphen (-), deleted nucleotide; red small letters (+), inserted nucleotide.

Table S2. Primers used in targeted deep sequencing for off target (OT) analysis.

Primer Name	Sequence
OT1 F	5′-GTCATATGGGTGCATAGTGAGAC-3′
OT1 R	5′-ATCACGATCAGCAGTCCATGA-3′
OT1 F1	5′-TTCATTAACCTATCGCACTCC-3′
OT1 R1	5′-GGTCCCAGAACAGTATCCA-3′
OT2 F	5′-CACTATCTCAAGAAGCTCTGCC-3′
OT2 R	5′-AATTGATGGGAAGAAGAGTGTGT-3′
OT2 F1	5′-AAGAACCAAACACATAGAGAAGA-3′
OT2 R1	5′-CCTACTTGAGCTGGAAACC-3′
OT3 F	5′-TGTGGTTCAGAAATTGTGCGAG-3′
OT3 R	5′-GGGTCAAAAAGCACGTCACC-3′
OT3 F1	5′-GCAATCCTCCAAATAACCCT-3′
OT3 R1	5′-TGAATCCTCAAAACCCAAGAA-3′
OT4 F	5′-GAGTTTTGAGTGGTTTAAATGGT-3′
OT4 R	5′-CCACGGGATATAGGGGTCTA-3′
OT4 F1	5′-CATGCAAGGTAAACGGGAAT-3′
OT4 R1	5′-GCAAGCAATGTCTTTAGTAAACC-3′
OT5 F	5′-CACTAAGCCCAAGTCTGATGTAC-3′
OT5 R	5′-GCTCATCCACCAAGTACTCTC-3′
OT5 F1	5′-ACGCTCTCAGATTGGCTAG-3′
OT5 R1	5′-CGAAATAGGCCTCACACGG-3′
OT6 F	5′-GAAACGAGGAGAAGAATGGATGC-3′
OT6 R	5′-CACATTCTTTTGCTGTCTCTTCC-3′
OT6 F1	5′-TGGTTCCACCACAAGGAGAT-3′
OT6 R1	5′-ACTGAAGGCGCTTGTCAC-3′

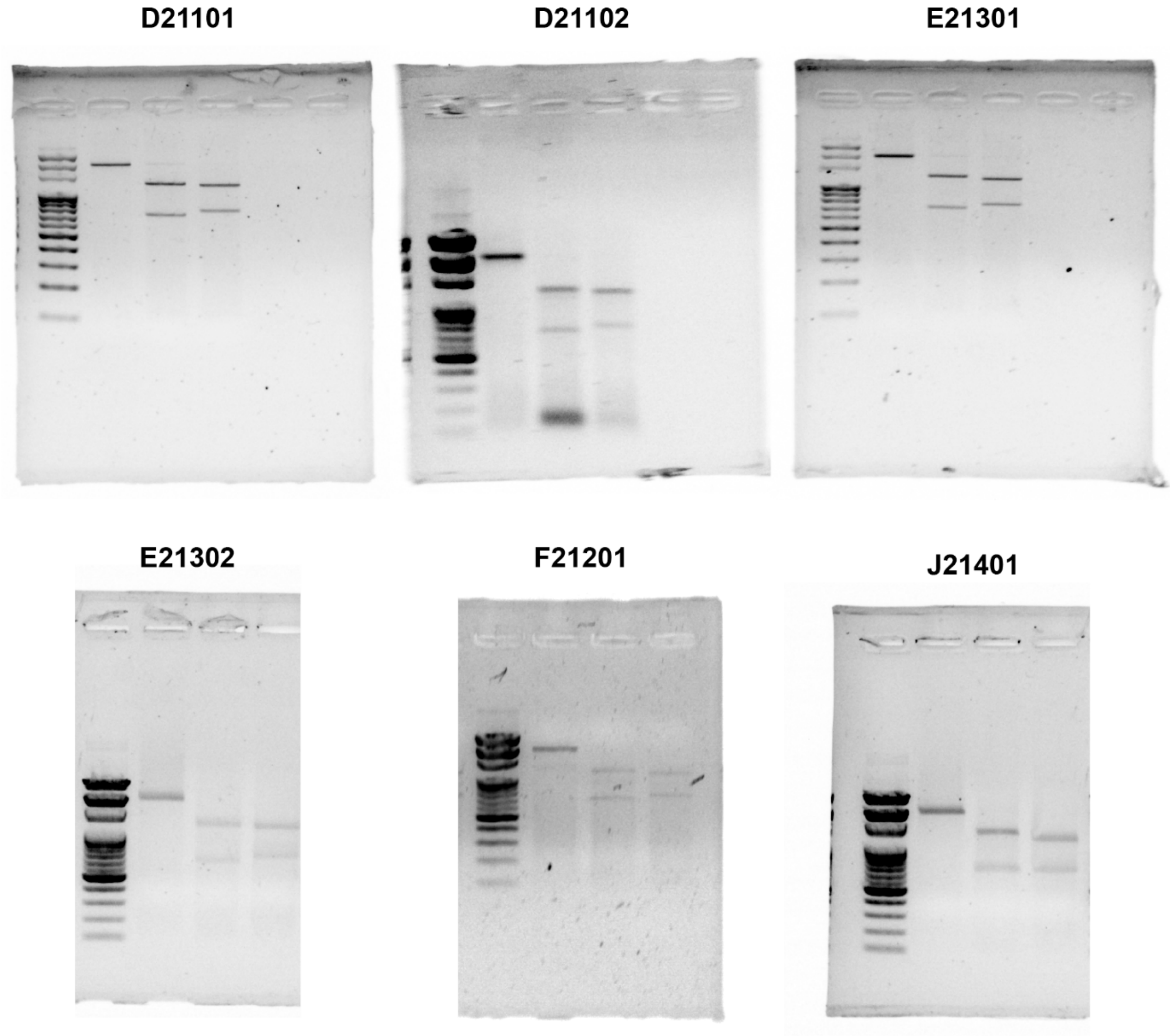

Figure S2. The original Gels of Figure 2.
